# Biliary adenofibroma: a precursor lesion of intrahepatic cholangiocarcinoma

**DOI:** 10.4322/acr.2023.453

**Published:** 2023-11-13

**Authors:** Mayur Parkhi, Rashmi Joshi, Manish Kumar, Aditi Sharma, Suvradeep Mitra, Lileswar Kaman

**Affiliations:** 1 Post Graduate Institute and Medical Education and Research, Department of Histopathology, Chandigarh, India; 2 Post Graduate Institute and Medical Education and Research, Department of General Surgery, Chandigarh, India

**Keywords:** Liver, Biliary Tract, Adenofibroma, Cholangiocarcinoma

## Abstract

Biliary adenofibroma (BAF) is an uncommon liver tumor with a high propensity for malignant transformation. The histomorphology of BAF with malignant transformation can show a spectrum of changes ranging from benign, dysplastic to frank malignancy. Thus, the diagnosis of BAF imposes the pursuit of dysplasia/ malignancy focus. We presented a case of intrahepatic cholangiocarcinoma arising from BAF in a 49-year-old woman with detailed histomorphology. We also performed a PubMed database search and tabulated all previously reported cases of BAF with dysplasia/ malignant transformation. A statistic comparison of age, sex ratio, size of the tumor, and survival following complete resection between BAFs with and without dysplasia/ malignancy from the retrieved data is presented. Our analysis did not highlight any statistically significant difference between BAFs with and without dysplasia/ malignancy in age, sex ratio, tumor size, and survival following complete surgical resection. Our study highlights the histopathology and immunohistochemistry of a case of BAF with malignant transformation and highlights the importance of this diagnosis in management. Further longitudinal studies on a larger cohort of patients are required to validate our findings.

## INTRODUCTION

Biliary adenofibroma (BAF) is an uncommon tumor arising from the bile ducts of the liver.^1^ Scarce data on its incidence, long-term outcomes, and optimal management is available due to its rarity. BAF is incidentally found during medical imaging studies such as ultrasound, computed tomography (CT), or magnetic resonance imaging (MRI) performed for unrelated reasons. Both hepatic lobes can be affected, and the tumor shows a slight female preponderance. The prognosis is excellent following complete surgical removal; however, there is a high risk of recurrence in case of incomplete excision.^2^ Although biologically benign, BAF can show dysplastic changes or malignant transformation, thus serving as a precursor lesion for intrahepatic cholangiocarcinoma (ICC). More than half of the reported cases of BAF showed malignant transformation.^2-17^ Herein, we document an uncommon case of BAF with microscopic foci of malignant transformation into ICC. We thoroughly searched the PubMed database for similar cases and briefly reviewed the literature.

## CASE PRESENTATION

**Figure 1:**
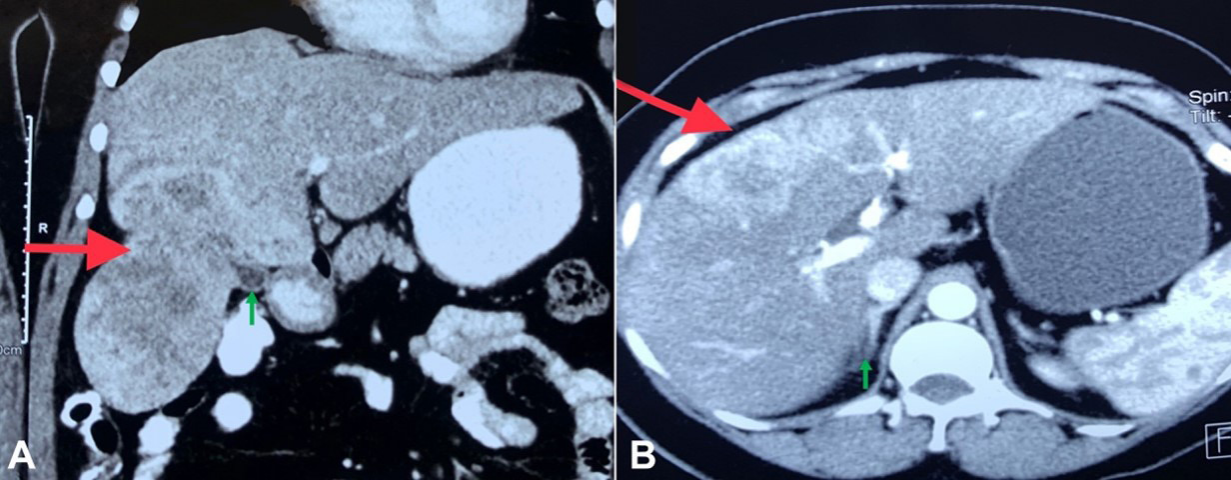
**A** and **B –** Abdominal CECT Imaging shows a hypodense heterogeneous mass lesion (red arrow) in relation to the gallbladder (green arrow).

A 49-year-old hypertensive, non-diabetic female patient complained of pain in the right upper quadrant and epigastric region for the last 8 months. The pain was insidious in onset, mild to moderate in intensity, dull aching, and intermittent in occurrence. She also complained of bloating sensation and non-projectile vomiting. Abdominal ultrasonography showed a hypoechoic mass measuring 10x7 cm in the right lobe of the liver with increased vascularity. The CECT showed an enlarged liver (17cm span) with an ill-defined mild hypodense lesion in segments IV and V, abutting and engulfing the fundus of the gallbladder (Figures 1A and 1B). The possibility of gallbladder carcinoma could not be excluded. The PET-CT revealed FDG-avid heterogeneously enhancing mass in the segments IVb, V, and VI of the liver (SUV^max^ 11.5). The patient underwent en-bloc mass resection with cholecystectomy. The intraoperative frozen section suggested the possibility of BAF. Later, the whole specimen was subjected to histological examination.

**Figure 2:**
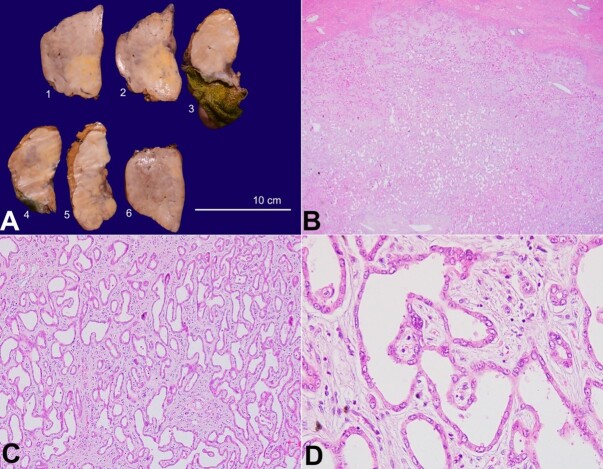
Photomicrographs of the tumor **A –** Gross picture of the mass highlighting a solid, homogeneous, and fleshy liver mass with an intact gallbladder; **B –** Histomorphology of the benign area of BAF highlighting well-circumscribed but non-encapsulated mass (H&E; 40x); **C –** The tumor is arranged in irregular glands embedded in the hyalinized stroma (H&E; 100x); **D –** The glands are lined by flat cuboidal non-mucin-secreting epithelia (H&E; 400x).

The surgical specimen measured 17x7x6cm and showed a well-circumscribed, greyish-white, firm, and homogeneous fleshy mass measuring 8x9x5cm in a non-cirrhotic background. The attached gallbladder measured 8cm in its longest axis and showed a velvety mucosa (Figure 2A).

The liver resection margin was 0.3cm away from the mass. The histopathology revealed a predominantly circumscribed but non-encapsulated tumor arranged in acini, tubules, and cysts embedded in an abundant fibrotic stroma. The acini were lined by a single layer of non-mucin-secreting epithelia with round nuclei, vesicular chromatin, inconspicuous nucleoli, and eosinophilic to amphophilic cytoplasm (Figures 2B to 2D). Focal apocrine snouts were noted. 

**Figure 3:**
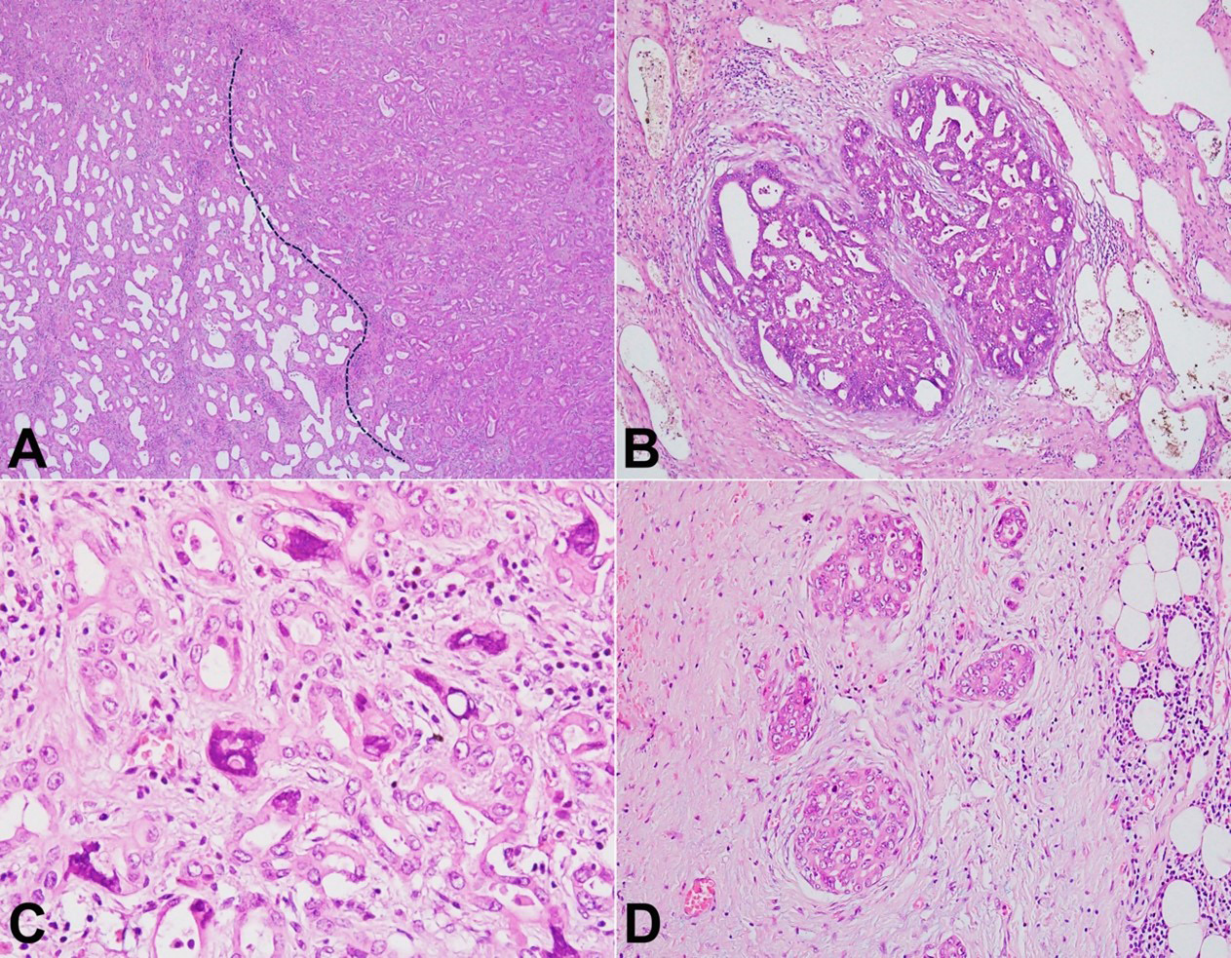
Photomicrograph of the tumor. **A –** Scanner view of the BAF showing the transition from the benign area (left side of the dotted line) to the dysplastic/malignant area (right side of the dotted line), the malignant area showing overcrowding and minimal stroma (H&E; 40x). **B** to **D –** Malignant and gallbladder-bed-infiltrating foci of BAF highlighting cribriform pattern (B; H&E, 100x), marked pleomorphism (C; H&E, 400x), and infiltrating nests single cells (D; H&E, 200x).

Multiple foci exhibited cytoarchitectural dysplasia. The architectural changes in the dysplastic/ malignant foci were noted in the form of structural complexity, solidification, and cribriform pattern with a marked reduction in the intervening stroma, while the cytology showed nuclear pleomorphism, enlargement, stratification, overcrowding, and hyperchromasia (Figures 3A to 3C). These foci of dysplasia, brisk mitoses, foci of necrosis, and infiltration of the gallbladder bed in the form of tiny nests, single cells, and islands favored a malignant transformation (Figure 3D).

**Figure 4:**
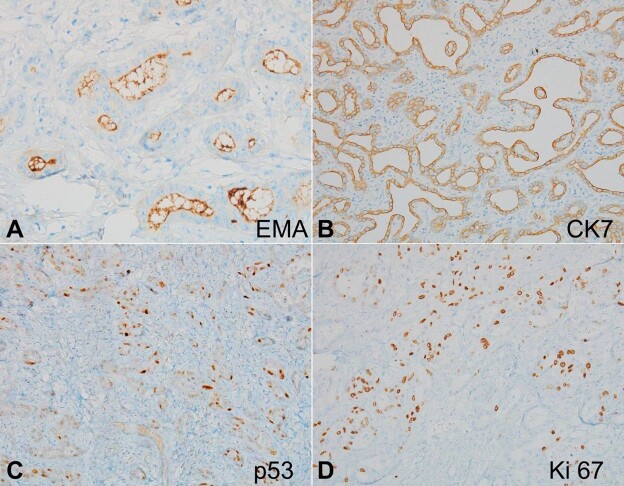
Photomicrograph of the tumor. **A** to **C –** The malignant focus shows the expression for EMA (A; 400x), CK7 (B; 200x), and p53 (C; 400x). **D –** Ki-67 labeling index is high in the malignant focus (400x).

The tumor cells in both benign and malignant foci were immunopositive for EMA (Figure 4A) and CK7 (Figure 4B). High p53 (Figure 4C) immunostaining was noted in the malignant area. Besides, Ki-67 labeling indices in the benign and malignant areas were 1-2% and 30-40% (Figure 4D), respectively. The background liver was non-cirrhotic showing centrilobular macrovesicular steatosis and mild porto-lobular inflammation. Thus, a diagnosis of adenocarcinoma (intrahepatic cholangiocarcinoma (ICC)) arising in the background of BAF was rendered. The omental tissue, hepatoduodenal ligament tissue, periportal, and posterior pancreaticoduodenal lymph nodes were tumor-free.

## DISCUSSION

Biliary adenofibroma (BAF) is an uncommon primary solid-cystic hepatic benign epithelial neoplasm. In 1993, Tsui et al.^1^ were the first to document this entity as a benign neoplasm in a 74-year-old lady. It is often asymptomatic; however, some patients may present with abdominal pain, nausea, or jaundice. In 2001, the potential of dysplastic change and/or malignant transformation was published.^5^ The chances of missing microscopic foci of invasion occur in cases undergoing incomplete tissue sampling at the time of surgery/ intraoperative frozen section.

We diligently searched the PubMed database for cases of BAF and came across only about 37 cases of BAF, including individual anecdotes and case series.^1-7^ All anecdotal cases of BAF documented in the English literature were reviewed and variable parameters like the age, sex, size of the tumor at the time of diagnosis, and survival following resection were compared by appropriate statistical tests among BAF, BAF with dysplasia, and BAF with malignant transformation categories. We found 26 cases of BAF with dysplasia (n=9)/ malignant transformation (n=17) documented in 22 studies, including the index case (Table 1).^2-18^

**Table 1:** The clinico-pathological data of biliary adenofibroma showing features of dysplasia and malignant transformation reported in the English literature

ref	Age	Sex	Tumor size cm	Hepatic Site	Microscopy	Background liver	Metastasis	Recurrence	FU
^5^	21	M	20	RL	BAF with MT	NA	No	No	2 years; Alive
^6^	25	M	20c	RL	BAF with MT	NA	Lung	Yes	3 yrs; Alive
^7^	53	F	6.5x5	LL	BAF with MT	NA	No	No	1 yrs; Alive
^17^	40	M	7x6	RL	BAF with D	Fulminant Hepatitis B	No	No	8 months; Died
^18^	69	F	3.3x2.8	RL	BAF with D	Normal	No	No	4 yrs; Alive
^8^	77	M	4x3	LL	BAF with MT	Portal inflammation, focal steatosis	No	NA	NA
^19^	57	F	11.8x7.5	RL and LL	BAF with D	NA	No	No	5 yrs; Alive
^9^	71	F	5.7	LL	BAF with MT	Von Meyenburg complex	No	No	NA; Alive
^10^	71	M	14.5x10.6.3	LL	BAF with MT	NA	No	No	9 yrs; Dead
	71	M	6.6x6.3	CL	BAF with MT	NA	No	No	1 month; Alive
^11^	37	F	4.9x4.8x4.6	RL	BAF with MT	NA	No	No	5 days; Alive
^2^	47	F	16	RL and LL	BAF with D	NA	No	Yes	7.5 yrs; Died
	57	F	Right 10, 2.5 Left 1.7	RL and LL (multifocal)	BAF with D	NA	No	No	3 yrs; Alive
	70	F	12	RL	BAF with D	NA	No	Yes	3 yrs; Alive
	46	M	15	LL	BAF with D	NA	No	No	21 yrs; Alive
^12^	66	F	2	LL	BAF with MT	Chronic Hepatitis (HCV)	No	No	6 wks; Alive
^13^	63	F	6.3x5	LL	BAF with MT	NA	No	No	2 yrs; Alive
^14^	44	F	9	RL	BAF with MT	Chronic Hepatitis (HBV)	No	No	4 yrs; Alive
^20^	30	F	9.8x8.1x7.1	LL	Atypical BAF	NA	No	No	6 months; Alive
^3^	51	F	9.2x5.9	RL	BAF with MT	NA	No	No	1 yrs; Alive
^15^	63	M	15.5×9.6×14.2	RL	BAF with MT	NA	Yes	No	3 months; Alive
^16^	67	M	0.7-1.6 cm (multiple)	RL	BAF with dysplasia	Non-cirrhotic	No	No	2 months; Alive
^4^	64	F	1.5x1.5	RL	BAF with MT	HBsAg positive	No	No	NA
^21^	37	F	3.8x2.6x2.7	RL	BAF with MT	NA	No	No	Close follow-up advised; Alive
^22^	64	F	4.5x4.5x3	CL	BAF with MT	NA	No	No	3 years; Alive
IC	49	F	10x7	RL	BAF with MT	Non-cirrhotic	No	No	3 months; Alive

BAF, biliary adenofibroma; CL, caudate lobe; D, dysplasia; F, female; IC= index case; LL, left lobe; M, male; max., maximum; MT, malignant transformation; NA, not available; RL, right lobe; yrs, years; wks, weeks.

Surprisingly, BAF with dysplastic foci showed an incidental association with adenocarcinoma of the gallbladder in another case.^18^ The median age of presentation of BAF with dysplasia/ malignancy was 63 years (age range: 21 -77 years) compared to 68 years (age range: 38 – 83 years) in BAF without any dysplasia/ malignancy (no significant statistical difference). There was a slight female preponderance with male to female ratio of 1:1.8 in BAF with dysplasia/ malignancy (no significant statistical difference). Both hepatic lobes were almost equally involved. Rarely the caudate lobe also acted as a primary site.^10^ BAFs mostly presented as a solitary mass, but multifocality can also occur.^2,18^ There was significant variability in the mass size ranging from 0.7cm to 20 cm. No significant difference in size was noted between BAFs and BAF with dysplasia/ malignancy. Grossly, BAF presented as well-circumscribed but unencapsulated mass lesions, with solid and cystic areas in variable amounts. The cystic areas mostly appeared microcystic (sponge-like); however, macrocystic spaces (>1cm) can also be seen.

On light microscopy, BAF is a well-circumscribed lesion composed of characteristic epithelial and stromal components in variable amounts. The epithelial element shows the arrangement in glands, tubules, and microcysts, which are lined by non-mucinous cuboidal to low columnar epithelium. In rare cases, apocrine change is also seen.^4,19^ The nuclei are cytologically benign, and mitotically, they are inactive. The stroma is fibrous and paucicellular, with no significant inflammation. The literature review showed that nearly 70% of BAF cases showed dysplasia/ malignancy.^2-22^ Rare ones can show microinvasive components (<1mm).^8^ The invasive front is usually seen as a pushing margin or as single-cell infiltration. Perineural and lymphovascular invasion may be noted less often.^9^ On immunohistochemistry, the lining epithelium shows immunoreactivity for EMA, CK7, CK19, and CA19-9 indicating the biliary phenotype. The reported Ki-67 proliferating index is less than 10% in BAF, whereas 20-30% in the invasive component.^4,9,12-15^

Tumor surgical removal is the standard treatment for BAF, and the prognosis is generally excellent, with a low risk of recurrence.^2^ The prognosis appears good even in the presence of dysplasia/ malignancy after the mass complete excision (Table 1). This contrasts with the relatively poor prognosis of ICC, although the available data is limited. We could not document any significant difference between the survival of BAFs and BAFs with dysplasia/ malignancy following complete surgical resection. The optimal management of this rare tumor needs to be well-established due to the lack of large-scale studies and limited clinical experience. Therefore, careful monitoring of patients after surgery is crucial to detect any potential recurrence or complications.

In conclusion, the diagnosis of BAF imparts two-fold significance. First, the diagnosis necessitates a thorough search in the pursuit of dysplastic focus and/ or invasiveness. Secondly, the diagnosis of an intrahepatic cholangiocarcinoma alludes to a diligent search for a component of BAF as the prognosis appears to be different between an intrahepatic cholangiocarcinoma, not otherwise specified, and an intrahepatic cholangiocarcinoma arising in the background of BAF, the former having a worse one. However, this statement is not validated due to the rarity of the latter entity. Therefore, complete surgical excision in BAF and wide local excision in case of associated invasive carcinoma is recommended. Incomplete resection or positive surgical margins, or multifocality may lead to recurrence.
